# Effects of leucine-enriched essential amino acid supplementation on muscular fatigue and inflammatory cytokines in wheelchair basketball players

**DOI:** 10.20463/pan.2020.0013

**Published:** 2020-06-30

**Authors:** Young Hwan An, Jisu Kim, Hee-Jae Kim, Kiwon Lim

**Affiliations:** 1 Department of Physical Education, Konkuk University, Seoul Republic of Korea; 2 Department of Sports Medicine and Science in Graduate School, Konkuk University, Seoul Republic of Korea; 3 Physical Activity and Performance Institute, Konkuk University, Seoul Republic of Korea

**Keywords:** Wheelchair basketball, muscular fatigue, inflammatory cytokine, leucine-enriched essential amino acid mixture, disabled athletes

## Abstract

**[Purpose]:**

This study aimed to investigate the effects of leucine-enriched essential amino acid (LEAA) supplementation on muscle fatigue and the level of inflammatory cytokines in wheelchair basketball players after a basketball game and interval training.

**[Methods]:**

Of the ten recruited wheelchair basketball players (aged 34.5±8.9 years; lean body mass of 34.3±10.0 kg) who had spinal cord injury (SCI) and had undergone amputation, nine participated in the final test. These nine athletes received LEAA supplements (3 times 4.0 g/day) or placebo treatment in a double-blind, randomized, crossover study. We measured variables related to muscular fatigue and inflammatory response before the intense exercise and 4 days after recovery.

**[Results]:**

The significant effect of LEAA supplementation was inhibition of circulating IL-6 levels in the LEAA-treated group compared with the placebo group (P < .05). However, no changes were observed in the TNF-α and creatinine kinase levels. Moreover, analysis of variance analysis showed no significant difference in the relative values of muscle soreness. However, the effect size analysis with Cohen’s d reported a significant improvement in the relative values of whole body and back muscle soreness.

**[Conclusion]:**

Our results revealed that LEAA supplementation before and after intense exercise could help reduce muscle soreness and IL-6 levels in wheelchair basketball players.

## INTRODUCTION

People with disabilities are at risk of developing secondary health conditions, including high morbidity and mortality^1^. A sedentary lifestyle occurring soon after spinal cord injury (SCI) may be in contrast to an active physical engagement preinjury and therefore can be associated with profound physical deconditioning sustained throughout the lifespan^2^. SCI results in an accelerated trajectory of diseases and disorders that resemble those experienced with aging^2,3^. Characteristic conditions of this accelerated state include vascular diseases, arterial circulatory insufficiency, atherogenic dyslipidemia, type 2 diabetes, bone and joint diseases and related endocrine disorders, immune dysfunction, and pain of musculoskeletal and neuropathic origins^4-8^. To overcome these problems, various exercises have been devised to maintain the health of disabled people^9,10^.

Wheelchair basketball, a typical sport played while in a wheelchair, is similar to regular basketball^11^. Wheelchair basketball is designed for individuals with physical disabilities such as paralysis, amputation, SCI, or joint and musculoskeletal disorders^12^. Wheelchair basketball players have to perform highly intense movements, including starting and stopping the wheelchair and turning in different directions using upper body muscles only^13^. Because they only use small muscles (biceps, back muscles, brachial muscles, etc.) of their upper body while playing, they are not only at risk of early muscle damage and inflammatory response but also of more injuries as compared to regular basketball players who use both upper and lower body muscles at the same time^14^. Therefore, it is very important for a wheelchair basketball player to prevent early muscle damage and inflammation response and to recover quickly. Prolonged high-intensity exercises are known to induce oxidative stress, fatigue, and tissue injuries such as muscle damage indicated by an increase in creatine kinase (CK) and lactic dehydrogenase (LDH) levels^15^. Moreover, acute or intense exercise alters the systemic balance of cytokines which are cell signaling molecules associated with oxidative stress^16^. Cytokine is a type of protein secreted from immune cells such as T cells, B cells, and macrophages, and is involved in the transmission of signals between cells, control of cell behavior, and immune response. After exercise, initiation of muscle-tissue injury by high-force eccentric contractions promotes infiltration by inflammatory cells. These cells, in conjunction with local muscles, endothelial cells, and satellite cells, produce an array of cytokines to regulate the inflammatory process, including tumor necrosis factor-α (TNF-α) and interleukin-6 (IL-6)^17^.

IL-6 is synthesized and secreted by contraction of skeletal muscle fibers especially during exercise and is the first cytokine released in the circulation^18^. The serum levels of IL-6 have been found to rise rapidly during prolonged and high-intensity exercise followed by a complete decline to baseline levels thereafter^19^. The response of IL-6 to exercise has been reviewed as an indicator of muscle damage and a marked increase in circulating levels of IL-6 after intense exercise is a remarkably consistent finding in several studies^17-21^.

After intensive exercise, reactions that are similar to those caused from infections and injuries could appear in the human body. It has been reported that the blood plasma concentrations of various substances, such as cytokines like TNF-α and IL-6 that affect the function of white blood cells, could increase after intensive exercise^22,23^. Many researchers believe that the most important variable affecting immune and inflammatory responses is the intensity of the exercise. But reports describing how the muscle damage caused from exercise could be restored are not available^24^.

Previous studies reported that fatigue and muscle damage caused by exercise could be attenuated with adequate rest and nutrition as skeletal muscle adapted with training^18^. Therefore, many athletes have been using dietary supplements to prevent fatigue and muscle damage.

Branched-chain amino acids (BCAAs) are usually consumed as nutritional supplements by many athletes and people involved in regular and moderate physical activities regardless of their practice level. Unlike other amino acids, BCAAs, which consist of leucine, isoleucine, and valine, can be involved in energy production, an important aspect of muscle metabolism^25^.

Leucine is well known for stimulating mammalian target of rapamycin (mTOR), also known as a potent stimulator of protein synthesis^26^. Several studies have demonstrated that mTOR is not only a key pathway in inflammation-dependent muscle regeneration, but overexpression of mTOR has been found to reduce inflammation in cardiomyocytes and prevent cardiac dysfunction^27^. Moreover, a potent mTOR inhibitor, rapamycin, impairs muscle regeneration after injury^28^. Accordingly, some reports have also shown that the intake of leucine-enriched essential amino acid (LEAA) enhances recovery from fatigue and muscle repair by modulating inflammation^29,31^.

Despite these positive effects of LEAA, previous studies mainly evaluated the effects of LEAA on athletes without disabilities. Thus, we investigated the effects of LEAA ingestion on the level of muscle damage, inflammation, and delayed-onset muscle soreness (DOMS) in wheelchair basketball players after a basketball game and interval training.

## METHODS

### Participants

The participants’ characteristics are shown in Table 1. Ten male wheelchair basketball athletes with SCI and amputation participated in this study. The participants were selected from top-ranking teams in the Korean Paralympic wheelchair basketball league. The study design, purpose, and possible risks were explained to each participant before written consent was obtained. Out of the ten athletes, nine participated in the final test, while one athlete was not able to participate due to injury. All participants were restricted from engaging in vigorous physical exercises prior to the experiments. They were also strongly requested to continue their regular dietary habits, especially the intake of protein and food supplement during the experiments. All experiments were approved by the institutional review board of Konkuk University (IRB no.: 7001355-201606-HR-124).

**Table 1.** Individual anthropometric data, lesion levels, and point values.

Participants (N=9)	Age (years)	Height (cm)	Weight (kg)	BMI (kg/m^2^)	Body fat (%)	Lesion levels	Point values*
1	40	177.8	65	20.6	11.7	Amputation	4.5
2	28	179.7	68	21.1	15.6	SCI 12	3.0
3	24	180.4	102	31.3	13.0	Amputation	4.0
4	36	163.0	55	20.7	19.8	SCI 10	2.0
5	37	177.3	83	26.4	28.0	SCI 6	1.0
6	28	170.0	53	18.3	18.8	SCI	2.0
7	33	181.0	83	25.2	21.1	Polio	4.0
8	23	173.0	70	23.4	25.9	SCI 11	2.0
9	37	178.7	95	29.7	23.4	SCI 7	1.0
M±SD	34.5±8.9	175.6±5.9	74.8±16.9	24.0±4.42	18.6±4.95	-	-

All values are presented as mean ± standard deviation. BMI, Body mass index; SCI; Spinal cord injury. * Point value for each player: 1.0–4.5 (highest to least impact of impairment).

### Leucine-enriched essential amino acid supplement (LEAA supplement)

The LEAA supplement (Ajinomoto Co., Inc., Tokyo, Japan) contained 4.0 g of a mixture of 9 essential amino acids (leucine, 1.61 g; lysine, 0.67 g; valine, 0.44 g; isoleucine, 0.43 g; threonine, 0.37 g; phenylalanine, 0.27 g; methionine, 0.13 g; histidine, 0.07 g; and tryptophan, 0.03 g) per pack. By contrast, the placebo contained 4.0 g of maltodextrin per pack. Both supplements were processed such that they were indistinguishable based on their external appearance and taste. The composition of LEAA was based on previous studies, which reported that LEAA promotes muscle protein synthesis when ingested^32,33^. The amount of LEAA (3 times of 4.0 g/day), timing, and duration of ingestion were based on a previous study, which revealed that ingestion of supplements containing 12 amino acids attenuated muscle damage after exercise^27^. The supplements were ingested along with 200 ml of water.

### Experimental design

**Figure 1.**
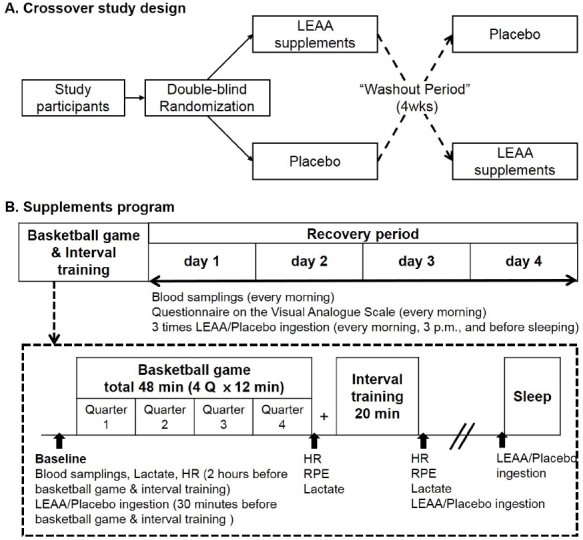
Experimental design.

The research design is shown in Figure 1. All participants were exposed to LEAA or placebo treatment in a double-blind, randomized, crossover experiment. The experiment was conducted in two 5-day phases, with a 4-week washout period. On the first day, the participants were involved in a four 12-min quarter basketball game and interval training, five 28-m sprints, five 28-m 4-step go and stop run, and three forward and backward run with one player. Within the 4-day recovery period, mild training programs were conducted. Exercise intensity was assessed after the basketball game and interval training by monitoring heart rate (Polar, Stanford, CT, USA), ratings of perceived exertion, and lactate levels (Lactate Pro 2, Arkray, Tokyo, Japan). Exercise programs were same in both phases.

Blood samples were collected every morning after at least 8 h of fasting. Samples were collected on the first day of each period. On the first day, all participants performed vigorous physical exercises and took LEAA or placebo 30 min before the game, after the interval training, and between dinner and bedtime. For the rest of the experiment period, the participants took LEAA or placebo every morning, at 3 pm, and before bedtime. The placebo was exactly same in shape and weight, indistinguishable from the LEAA supplement.

### Blood sample analysis

All study participants were restricted from drinking and engaging in intense physical activity 48 h prior to the study. They also fasted for 8 h, except for fluid intake, before the blood was drawn. Approximately 10 ml of blood was drawn using a disposable syringe and collected into two 5-ml SST and ethylenediaminetetraacetic acid vacutainer tubes. The blood samples were spun in the centrifuge for 15 min at the speed of 3000 rpm right after the collection. Then, the separated serum and plasma were kept at −70 °C until further use. Muscle damage was evaluated based on plasma CK levels (Bioclin Diagnostics, Sao Paulo, Brazil), which was performed according to the method established by Oliver^34^. To evaluate inflammatory cytokine levels, IL-6 and TNF-α levels were measured with enzyme immunoassay method using ELx50 (an automatic washing system) and SIRIO-S (a microplate photometer equipped with a 450 nm/620 nm filter, BioTek Inc., USA). The reagents used to measure IL-6 and TNF-α high sensitivity were IL-6 (Bender MedSystem, Germany) and TNF-α (Bender MedSystem, Germany).

### Muscle soreness

Muscle soreness was measured using visual analogue scale (VAS), a valid and reliable pain measurement methods proven by many previous studies^35,36,37^. On a 100-mm horizontal line, a 0 measurement on the left end means no pain at all, while a 100 measurement on the right end means maximum pain. In the study, participants were asked to indicate the measure of their pain on the VAS in six different areas of the body – whole body, forearm, upper arm, shoulder, back, and abdomen, 4 days after recovering from a basketball game and an interval training.

### Statistical analysis

All data are expressed as mean ± standard deviation. The change in values was calculated by subtracting the baseline value from that on each recovery days (days 1, 2, 3, and 4) in CK, IL-6, and TNF-α. The relative data was calculated by dividing the value for each recovery period by the day 1 value (VAS not measured at baseline) and then multiplied by 100 [e.g., day 3 = (day 3 / day 1) × 100]. A two-way (two groups × 5 recovery days) repeated measure analysis of variance (ANOVA) was used to determine the main and interaction effects among groups and the recovery days for mean inflammatory cytokine (IL-6 and TNF-α) and muscle damage (CK) change from baseline value, using SPSS version 21.0 (IBM, USA). The assumption of sphericity was tested by Mauchly’s test. If sphericity assumption was violated, the Greenhouse-Geisser correction was used. Post-hoc test used the least significant difference to evaluate the main and interaction effects. In the VAS, Cohen’s effect size was used since statistical significance mainly depends on the sample size. According to Cohen (1988), small = 0.2–0.5 is considered a small effect, 0.5–0.8 is medium, and 0.8–2.0 is large.

## RESULTS

### Heart rate, rating of perceived exertion, and lactate levels during exercise

In the study, the heart rate increased with the increase in workload during the exercise test (basketball play and interval training) in both trials (Table 2). The basketball game and interval training were performed with high intensity (approximately 150–164 bpm of 70%–80% of heart rate reserve; no data presented). No significant difference was observed in the mean heart rate (LEAA: 146.3 ± 6.0 bpm; placebo: 143.8 ± 3.9 bpm) and peak heart rate (LEAA: 182.1 ± 6.9 bpm; placebo: 176.3 ± 10.6 bpm) between the groups during the game. Moreover, no significant difference was observed in the mean heart rate (LEAA: 143.77±11.68 bpm; placebo: 139.50 ± 7.76 bpm) and peak heart rate (LEAA: 170.66 ± 6.70 bpm; placebo: 173.87 ± 5.48 bpm) between the LEAA and placebo trials during the interval training. The rating of perceived exertion was not significantly different between the LEAA and placebo groups during the game (LEAA: 14.44 ± 1.87; placebo: 13.89 ± 1.61) and during the interval training (LEAA: 16.88 ± 2.10; placebo: 16.67±1.87). There was no significant difference in the lactate concentrations between the LEAA and placebo groups during the resting phase (LEAA: 2.93 ± 1.59 mmol; placebo: 2.71 ± 1.39 mmol) after the game (LEAA: 5.42 ± 2.15 mmol; placebo: 5.92 ± 2.52 mmol), and after the interval training (LEAA: 11.04 ± 5.16 mmol; placebo: 9.78 ± 5.39 mmol).

**Table 2.** Exercise intensity during and after the basketball games and interval training.

Variables	Placebo (N=9)	LEAA (N=9)	*P*
HR rest (bpm)	65.1 ± 8.5	65.1 ± 8.5	.913
HR mean during game (bpm)	143.9 ± 4.0	143.9 ± 4.0	.343
HR peak during game (bpm)	176.4 ± 10.6	176.4 ± 10.6	.203
HR mean during interval (bpm)	139.5 ± 7.8	139.5 ± 7.8	.395
HR peak during interval (bpm)	173.9 ± 5.5	173.9 ± 5.5	.301
RPE during game	13.9 ± 1.6	13.9 ± 1.6	.511
RPE during interval	16.7 ± 1.9	16.7 ± 1.9	.832
Lactate rest (mmol)	2.7 ± 1.4	2.7 ± 1.4	.757
Lactate after game (mmol)	5.9 ± 2.5	5.9 ± 2.5	.657
Lactate after interval (mmol)	9.8 ± 5.4	9.8 ± 5.4	.621

All values are expressed as mean ± standard deviation. LEAAs, leucine-enriched essential amino acids, HR, heart rate; RPE, rating of perceived exertion—measured using the Borg scale.

### Blood parameter of muscle damage and inflammation

**Figure 2.**
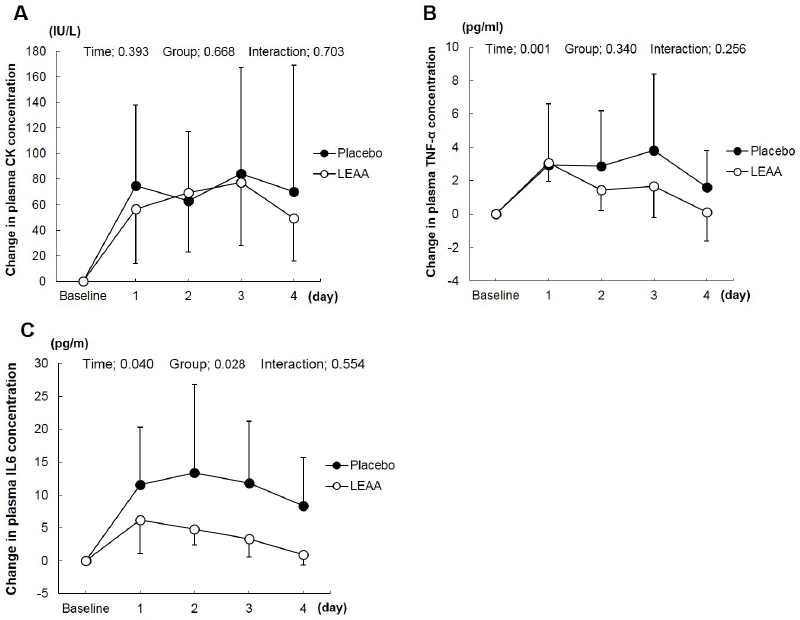
Changes in blood parameters of muscle damage and inflammation. Graph showing mean CK, TNF-α, and IL-6 from the baseline of the placebo and LEAA groups within the 4-day recovery period (after the basketball games and interval training). Data expressed as mean ± SD (n = 9 in each group). P values indicated using two-way repeated measures ANOVA.

The serum CK activities throughout the experimental period, which served as a blood parameters of muscle damage, are presented in Figure 2. On the first day of recovery, serum CK levels tended to be lower in the LEAA intake group than in the placebo group (p = .0.668). However, there was no significant difference in CK levels during the recovery period between the LEAA and placebo groups (days 1, 2, 3, and 4). CK levels peaked on recovery day 1 (LEAA: 88.9 ± 31.5 IU/L; placebo: 104.6 ± 41.9 IU/L) and day 3 (LEAA: 158.3 ± 58.7 IU/L; placebo: 167.3 ± 59.5 IU/L) in both groups after the basketball game and interval training.

The serum TNF-α and IL-6 levels, which served as blood parameters of inflammation, are presented in Figure 2.

The TNF-α in the LEAA group peaked on day 1 and rapidly decreased during the recovery period. On the contrary, the serum TNF-α levels in the placebo group peaked on day 3 and were higher than those in the LEAA group. However, there were no significant differences in TNF-α levels between the two groups (time: 0.001; group: 0.340; interaction: 0.256). Serum IL-6 was significantly increased after exercise in both LEAA and placebo groups. A two-way ANOVA with repeated measures for IL-6 showed that time had a significant effect (P = .040) on IL-6 levels (P = .028), while group-by-time interactions (P = .554) did not.

### DOMS following exercise

**Figure 3.**
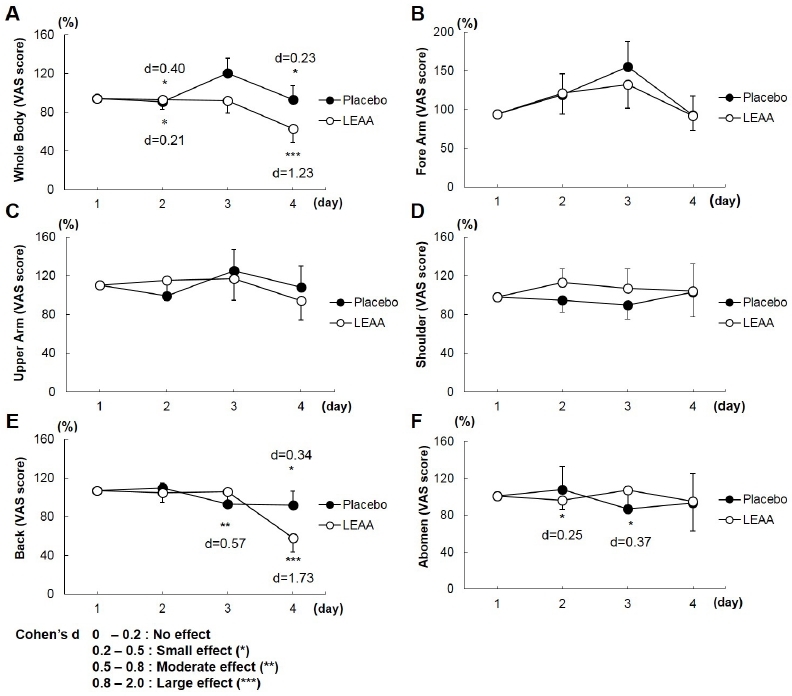
Visual analogue scale of muscle fatigue. Graph showing the changes in mean VAS values of the placebo and LEAA groups from day 1 within the 4-day recovery period (after the basketball games and interval training). Data expressed as mean ± SD (n = 9 in each group). Effect size indicated using Cohen’s d.

Figure 3 shows the VAS scores for subjective DOMS assessment. The VAS scores of all examined areas (whole body, forearm, upper arm, shoulder, back, and abdomen) had no significant difference between the LEAA and placebo groups with repeated measures ANOVA during the recovery period (days 1, 2, 3, and 4). However, the VAS scores in the whole body and back peaked on Day 1 and then rapidly decreased in the LEAA group, whereas the VAS scores in the placebo group peaked on days 2 and 3 and did not subside compared with those in the LEAA group. Furthermore, there was a significant improvement in the relative values for the whole body (day 1, d = 0.40; Day 4, d = 1.23) and back soreness (day 4, d = 1.73) as shown in the effect size analysis with Cohen’s d in the LEAA groups (Figure 3).

## DISCUSSION

The study was designed to identify the impact of LEAA ingestion on muscle damage and inflammatory response in disabled wheelchair basketball players. The present findings indicated that LEAA ingestion suppressed circulating IL-6 levels. Furthermore, LEAA improved the recovery of whole body and back soreness after the game/interval training within the 4-day recovery period. However, there was no significant difference between the TNF-α and CK levels.

Several studies have reported that CK is one of the markers of exercise-induced muscle damage, and in general, intense physical exercise is believed to increase CK levels in blood^38^. In our study, both groups showed an increase in CK levels from baseline after the wheelchair basketball game and interval training. On the first day of recovery, serum CK levels tended to be lower in the LEAA intake group than in the placebo group. This trend could be a factor that effectively reduces the CK level, increased during high-intensity exercise. However, compared with the placebo group, LEAA ingestion did not reduce the CK levels during the recovery period. Contrary to our results, Matsumoto K et al. reported that the plasma CK, LDH, and granulocyte elastase levels after the training program in the BCAA trial on 12 long-distance runners (20 ± 1 years) were lower than those in the placebo group (−21%, −6 %, and −15%, respectively)^14^. In addition, Kim DH et al. reported that BCAA ingestion reduced the CK and LDH levels after the cycle exercise (at 70% VO2 max) to exhaustion^39^. However, in this study, we could not detect the effectiveness of LEAA ingestion with regard to CK values. This is because the CK value varies greatly depending on the sampling point. According to a recently published review paper, it appeared that a high volume of upper body exercise with short rest intervals taken between sets was more likely to produce the greatest increase in CK levels^38^. In our study, total serum CK activity was markedly elevated 24 h after the exercise bout and gradually returned to baseline levels [40]. Although the participants were wheelchair basketball players who frequently used their upper body for movement and practice, CK samples were obtained 24 h after performing the exercise. In addition, there were two more possible reasons for the insufficient CK elevation. One might be the insufficient intensity of exercise and the other reason might be the fact that plasma CK in trained athletes is less likely to elevate by exercise. For these reasons, the effect of LEAA supplementation on CKs could not be confirmed in this study.

Cytokines control the immune and inflammatory response. Skeletal muscle inflammation can increase the expression and activity of factors such as TNF-α, ROS, and IL-6, which in turn could contribute to protein degradation and attenuate protein synthesis^41,42^. By contrast, BCAA supplementation could counteract such effects by suppressing skeletal muscle proteolysis and stimulating protein synthesis^43^. Our study revealed that LEAA attenuated IL-6 expression^15^. Nosaka et al. reported that resistance training with BCAA ingestion decreased the inflammatory response and muscle soreness^44^. Furthermore, Bassit et al. found that ingestion of BCAA (6.0 g of 60% L-leucine, 20% L-valine, and 20% L-isoleucine/day) decreased the inflammatory response and muscle soreness after the triathlon game (Olympic triathlon: a 1.5-km swim, a 40-km bike, and a 10-km run)^32^. The recent reports demonstrated mTOR as a key pathway in inflammation-dependent muscle regeneration^24^. Kato et al. reported that LEAA accelerated recovery from muscle damage by preventing excessive inflammation and IL-6 expression by stimulating the mTOR^31^. Additionally, Dreyer et al. and Pasiakos et al. reported that LEAA stimulated muscle protein synthesis after performing several types of exercises^30,45^. Therefore, LEAA may have sufficient biological activity to promote post-exercise recovery by increasing protein synthesis^29^.

The TNF-α values of the LEAA group were lower than those of the placebo group, but no significant differences were found. A large number of intense, eccentric muscle action during team sports can cause muscle damage of varying degrees and may trigger inflammatory responses. In the players of team sports, TNF-α levels are typically elevated^46^. The participants of this study were athletes who had SCI or amputation. As previously reported, they also had immune system disorders due to deterioration in white blood cell count^39,47^.BCAA or leucine amino acids suppress DOMS, which is a typical symptom of muscle damage^40^. DOMS is the pain felt in the skeletal muscles upon palpation or movement following exercise, which generally peaks within 24–48 h^48^. Shimomura Y et al. reported 12 young, untrained females who ingested 100 mg/kg body weight of BCAA before the squat exercise^49^. DOMS peaked on days 2 and 3 in both trials (BCAA and control; dextrin), and the level of soreness was significantly lowered in the BCAA trial than in the control group^50^. In our study, the prevalence of whole-body fatigue after the event was highest on day 2 and back on day 3 but was lower on day 4 in the LEAA group. The sense of fatigue was similar to that of muscle soreness after performing an exercise. Previous studies reported that muscle soreness was a typical consequence of eccentric exercise-induced muscle damage; however, the underlying mechanism of muscle soreness is not clearly understood. It is assumed as an inflammatory response to muscle damage^51^.

Despite several research to evaluate the effects of BCAA, no study has reported the effects of BCAA on patients with disabilities and healthy athletes. In recent years, researchers found that BCAA ingestion reduced muscle fiber disruption, resulting in lower peak values of serum IL-6 and CK after exercise training^52,53^. In particular, leucine, a component of BCAA, has been known to play the key role in activating the mTOR signaling pathway and inducing muscle synthesis. mTOR signaling pathway serves as a central regulator of cell growth and proliferation through protein synthesis. It is also known to control differentiation and fusion of skeletal muscles that may increase the size of the muscle cells^54^. Bolster et al.’s research on experimental rats exemplified that intense exercise significantly increased mTOR expression^55^. Ge et al. reported that the muscle regeneration process was regulated by the mTOR pathway. Kato et al. investigated the effects of LEAA, as a potent mTOR stimulator on muscle soreness^56^. Several studies have indicated that consuming L-leucine may significantly increase muscle protein synthesis and decrease proteolysis^57^. Therefore, recent studies have applied LEAAs to a variety of subjects including elderly and young individuals^47,52,58^ during exercise training, which indicates a dose-dependent effect of leucine on muscle protein synthesis. This is the first study to examine the effects of LEAA ingestion on muscle damage and muscle soreness in wheelchair basketball players. Future studies should be conducted to establish the BCAA intake capacity according to the level of disability or injury. In conclusion, LEAA ingestion may reduce muscle fatigue (DOMS) and inflammatory response (IL-6) during the recovery period after an intense exercise.

Despite the numerous studies conducted to evaluate the effects of LEAA, the effectiveness of LEAA in disabled athletes has not yet been examined. Our results revealed that LEAA supplementation before and after intense exercise session could help reduce muscle soreness and inflammatory response in wheelchair basketball players. Further studies are required to identify the mechanisms responsible for the effects of LEAA supplementation in disabled athletes.
